# Anterior Cruciate Ligament Injury and its Rehabilitation in Nepal

**DOI:** 10.31729/jnma.6884

**Published:** 2021-07-31

**Authors:** Suraj Bhusal, Ujjwal Dotel, Shamed Kumar Katila, Saugat Shrestha

**Affiliations:** 1 Orthoplast Rehabilitation and Sports Centre, Gwarko, Kathmandu, Nepal

**Keywords:** *anterior cruciate ligament*, *management*, *physiotherapy*, *rehabilitation*

## Abstract

Anterior cruciate ligament is one of the most common ligaments to get injured especially in athletic population. It is a band of dense connective tissue which arises from the anteromedial aspect of the intercondylar area on the tibial plateau and passes upwards and backwards to attach to the posteromedial aspect of the lateral femoral condyle. There is an increasing rate of its reported injuries among athletes in Nepal and surgeons report increased consultations among athletes regarding adequate intervention strategies. Factors like overuse, inadequate recovery time, playing surface, fitness incompatibility are involved in the increase of anterior cruciate ligament injury among athletes in Nepal. The treatment approach for anterior cruciate ligament injury is ever-evolving with regular studies and innovation, but constant guidance and rehabilitation in an institution-based setting have shown positive feedback in recovery and return to play.

## INTRODUCTION

The anterior cruciate ligament (ACL) is a band of dense connective tissue which attaches femur to the tibia in anterior joint line.^1^ We consider it as a key structure in the knee joint, as it resists anterior tibial translation and rotational loads.^1^ It is a band of dense connective tissue which arises from the antero-medial aspect of the inter condylar area on the tibial plateau and passes upwards and backwards to attach to the posteromedial aspect of the lateral femoral condyle.^1^ It is one of the most common ligaments injured, especially in athletes which can be career-ending. It occurs most frequently in those who play sports involving pivoting (e.g., football, basketball, volleyball, rugby, cricket, etc.). Injuries can range from mild (such as small tears/sprain) to severe (completely torn ligament). Both contact and noncontact injuries can occur, although non-contact tears and ruptures are most common.^3^

## PATHO-ANATOMY OF ACL INJURY

The ACL is comprised of two bundles named as per insertion sites on the tibia: anteromedial (AM) and posterolateral (PL), at the lateral wall of the intercondylar notch, two prominent osseous ridges marks the borders of the femoral ACL insertion site: the lateral intercondylar ridge demarcates the anterior border of the ACL, while the lateral bifurcate ridge, running perpendicular to the lateral intercondylar ridge, separates the femoral attachment sites of the two bundles.^2,3^ ACL is primary structure that restrains anterioposterior and rotatory stability of knee joint in lower flexion angles. Primary cause of ACL tear is restraints to anterior tibialdisplacement when the knee is kept at 90 degrees of flexion and secondary restraints to tibial rotation & varus-valgus angulation at full knee extension.^3-5^

## MECHANISM OF INJURY

### Non-contact Injury

If a person comes to a sudden stop or makes an abrupt change in direction, it may force the tibia to move forward causing a tear. This is known as non-contact injury. An example would be an athlete who comes to a sudden halt while running.^5,6^

### Contact Injury

When the knee is forced into hyperextension (e.g., during sports), during valgus deformation of knee, or due to head-on collision (e.g., during road traffic accidents), the ACL may tear or rupture which is termed as contact injury. These patients usually present with poly-trauma associated with other life-threatening injuries.^5,6^

## SCENARIO IN NEPAL

An ACL rupture is a serious injury that can be career-ending for athletes. A re-rupture after primary ACL reconstruction occurs in 1 % to 11 % of all athletes. Injuries to the ACL are relatively common knee injuries among athletes.^6^ It occurs most frequently in those who play sports involving pivoting (e.g., football, basketball, volleyball, futsal, rugby and even cricket).

In Nepal incidence of ACL injuries either it is tear or even a rupture is increasing rapidly. In present day, there have been increased cases of ACL rehabilitation following repair, reconstruction and even conservatively managed. In past 6 months there has been 6-14 documented case of rehabilitation following ACL reconstruction in a single rehabilitation center (Orthoplast Rehabilitation Center).

In our experience, athletes are unaware of the role of physiotherapy in sports and injury-prevention. Some might even be oblivious to the role physiotherapy plays in training, fitness, injury care, and rehabilitation following surgeries.

**Cause of increased incidence in Nepal**

There is a need to establish the current status and reasons of increasing injury rates in sports in comparison to previous years and how is rehabilitation trending in sports conditioning. As mentioned earlier, ACL injuries have increased in the last couple of years. Some reasons for this rise could be an increase in the number of sports competitions and tournaments, increased participation of female athletes in each sport, increase in the game time compared to recovery period. Another interesting reason could be a lack of knowledge on adequate training, diet planning, importance of warm-up and cool-down, or the impact of ACL in players.

A common problem we encounter is that the clients we treat have little knowledge on fitness regimen, do not have a well-planned training schedule, do not give importance to their nutrition, and do not opt for rehabilitation following any surgical intervention.

## THE ROLE OF SPORTS ORGANISATIONS

In our experience, athletes that are supported by organizations or sports clubs adhere to institution-based rehabilitation more than those who do not. When subsidised by these organisations, rehabilitation time is adequate, and complications like an incomplete range of motion (ROM) in knee, reduced strength of knee stabilizers, fear of avoidance to activities of daily living (ADL's), or mental and physical incompetence to return to sports are lower. Treatment approach for ACL is ever evolving with regular studies and innovation, but constant guidance and rehabilitation in an institution-based setting has shown positive feedback in recovery and return to play.

## TREATMENT AND TECHNIQUES OPTED IN NEPAL

ACL cases have been rising day by day in Nepal and surgeons report increased consultations among athletes regarding adequate intervention strategies. Surgeon's report, in previous year's awareness about ACL injury without anyone informing about the symptoms were limited whereas in the present day with peer review, social media, increased rehabilitation units in Nepal, consultation has increased for understanding management approach.

**Non-operative/Conservative management-** One of the least approached method that is poorly tolerated by both young active adults and in the skeletally immature ones. This often leads to recurrent instability and the development of osteochondral defect and meniscal injuries.^2–7–5^

**Repair-** Poor outcomes have been associated with repair in comparison to ACLR from various reports.^2^ Recently, there has been an increasing interest in ACL preservation as an option to perhaps better restore native ACL anatomy, biomechanics, and neurosensory function.^2,7,5^

**Reconstruction-** Most widely used method with higher success rate compared to other techniques by using grafts such as hamstring tendon (HT), bone-patellar tendon-bone (BPTB), or quadriceps tendon (QT) to reconstitute the anatomy and function of the native ACL.^7,5^ This tissue can be harvested from either patient (autograft) or from a cadaver (allograft).

## STANDARDIZED PRINCIPAL OF ACL REHABILITATION

Basic principal followed all over the world for the treatment of ACL is as follows:

- Gain good functional stability
- Repair muscle strength
- Reach the best possible functional level
- Decrease the risk for re-injury

Open chain and Closed chain exercises are managed accordingly in rehabilitation.^8-10^

## METHODS OF REHABILITATION

Structured rehabilitation of ACL ruptures is similar for any treatment option, either reconstruction or nonoperative management with rehabilitation programs including cryotherapy (ice), gravity-assisted motion or continuous passive motion (CPM), protective bracing, electrical neuromuscular stimulation, and exercises aimed for strengthening, balance, proprioception, and reducing inflammation.^11-13^

## REHABILITATION IN NEPAL

Rehabilitation in Nepal depends upon physiotherapy settings present, such as:

1. Hospital based setting provides In-patient service for 0-1 week post-op, followed by 2-3 weeks OPD basis or else weekly follow-up basis
2. Clinic based settings-long term rehabilitation is adhered to focusing on pain, range of motion and strength but lacks adequate tools and methods for sports specific rehabilitation.
3. Rehabilitation centre based setting-long term rehabilitation provider with adequate tools to return to sports, but rare in a single province.
4. Home based rehabilitation as many players also return to home after surgery and they are prescribed with home based exercise protocol with regular follow-ups.

## PROS AND CONS IN REHABILITATION

**Pros:**

1. Evidence based practised has emerged immensely in few years.
2. More equipped Sports Injury centres are being established.
3. Specific orthopaedic surgeons as Arthroscopic surgeons are performing sports injury surgeries in large numbers which itself is another scope of rehabilitation.
4. Consultations with arthroscopic surgeons has been rising, athletes are concerned about injury status and remedy to return to sports

**Cons:**

1. Lack of enough well equipped centres.
2. Transportation facilities.
3. Official authorities have less knowledge about rehabilitation that indeed is driving force in professional associations to guide players.

Hospital based rehabilitation approach has not been uniform for ACL rehabilitation as multispecialty hospitals tend to provide high equipped care whereas other hospitals provide follow-up based and home based care, private clinics provides long term rehabilitation but patient are unable to afford its cost.

Players are not getting aids or supports from clubs or country for 6-9 months of continuous rehabilitation.

There are very few sports rehabilitation center having regular contact with surgeons and physiotherapist working together under one project.

## WAYS FORWARD

In the last one year there have been few documented cases that have been successfully rehabilitated and returned to sports following six to nine months of intensive rehab and two months of field-based rehabilitation. Primary factors reported are multidisciplinary approach in ACL management, inadequate support to players from organizations or clubs that can help players financially to continue rehabilitation for required time span. One of the major things to rectify is establishment of sports specific rehabilitation centres that can provide evidence based, protocol-based approach in rehabilitation and return to sports.

## References

[1] Duthon VB, Barea C, Abrassart S, Fasel JH, Fritschy D, Menetrey J (2006). Anatomy of the anterior cruciate ligament.. Knee Surg Sports Traumatol Arthrosc..

[2] Papa JA (2012). Athletic and sport issues in musculoskeletal rehabilitation (Musculoskeletal Rehabilitation Series).. J Can Chiropr Assoc..

[3] Monk AP, Davies LJ, Hopewell S, Harris K, Beard DJ, Price AJ (2016). Surgical versus conservative interventions for treating anterior cruciate ligament injuries.. Cochrane Database Syst Rev..

[4] Raines BT, Naclerio E, Sherman SL (2017). Management of anterior cruciate ligament injury: what's in and what's out?. Indian J Orthop..

[5] Kiapour AM, Murray MM (2014). Basic science of anterior cruciate ligament injury and repair.. Bone Joint Res..

[6] Ryder S, Johnson R, Beynnon B, Ettlinger C (1997). Prevention of ACL injuries.. Journal of Sport Rehabilitation..

[7] Felice GS, Villegas C, Taylor S (2015). Anterior cruciate ligament preservation: early results of a novel arthroscopic technique for suture anchor primary anterior cruciate ligament repair.. Arthroscopy..

[8] Sherman MF, Lieber L, Bonamo JR, Podesta L, Reiter I (1991). The long-term followup of primary anterior cruciate ligament repair. Defining a rationale for augmentation.. Am J Sports Med..

[9] Millett PJ (2010). ACL reconstruction rehabilitation protocol.. Sports Medicine and Orthopaedic Surgery..

[10] Cavanaugh JT, Powers M (2017). ACL Rehabilitation progression: where are we now?. Curr Rev Musculoskelet Med..

[11] Ellman MB, Sherman SL, Forsythe B, LaPrade RF, Cole BJ, Bach BR (2015). Return to play following anterior cruciate ligament reconstruction.. J Am Acad Orthop Surg..

[12] Marx RG (2020). Latest trends in ACL reconstruction.. Sports Med Arthrosc Rev..

[13] Stannard JP, Sherman SL, Cook JL, Grauer JN (2018). Soft tissues about the knee.

